# Editorial: The Significance of Mitogenomics in Mycology

**DOI:** 10.3389/fmicb.2020.628579

**Published:** 2021-01-07

**Authors:** Tomasz Kulik, Anne D. Van Diepeningen, Georg Hausner

**Affiliations:** ^1^Department of Botany and Nature Protection, University of Warmia and Mazury in Olsztyn, Olsztyn, Poland; ^2^B.U. Biointeractions and Plant Health, Wageningen Plant Research, Wageningen University & Research, Wageningen, Netherlands; ^3^Department of Microbiology, University of Manitoba, Winnipeg, MB, Canada

**Keywords:** fungi, mitochondrion, mitochondrial DNA, homing endonuclease, evolution

Fungi are a diverse group of eukaryotic organisms, ranging from unicellular yeasts to multicellular filamentous microorganisms and mushrooms, with beneficial and harmful effects to human health and the environment (Wu et al., 2019). Fungal diversity encompasses a broad range of taxa and associated differences in morphology, ecology, and life history strategies (Hyde et al., 2019; Naranjo-Ortiz and Gabaldón, 2020).

Like other eukaryotes, fungi contain mitochondria that are responsible for ATP synthesis and perform many biological tasks in fungal cells ranging from the production of chemical energy to synthesis of protein co-factors, and fatty acid, heme, and iron-sulfur cluster biosynthesis. To date, however, most of our knowledge on mitochondrial function/dysfunction comes from studies on *Saccharomyces cerevisiae*, which shows a high tolerance to mutations inactivating oxidative phosphorylation and the loss of mtDNA (Malina et al., 2018). This is in contrast to filamentous fungi that are obligate aerobes and the presence of functional mtDNA is essential.

The mitochondrial respiration chain has been linked to adaptation of fungi to oxygen-limited conditions or oxidative stress during certain phases of their life cycle, which explains the ability of fungi to survive in different environments and their potential to shift to different modes of living (Marcet-Houben et al., 2009; Grahl et al., 2012). Besides coping with oxidative stress, fungal pathogenicity, virulence, and mycotoxin production have been linked to mitochondrial morphology, integrity (Tang et al., 2018) and dynamics (Neubauer et al., 2015), and fission and fusion of mitochondria (Verma et al., 2018) and their overall implication in a range of vital cellular functions appear to be the common denominators underlying these processes (Medina et al.).

Mitochondria have been found to confer drug tolerance in both human and plant fungal pathogens. The mechanisms of resistance are complex and can occur at many different levels, however lipid homeostasis in mitochondria (Shingu-Vazquez and Traven, 2011) and mutations in genes encoding proteins that are involved in mitochondrial processes (Mosbach et al., 2017) appear to play the most important role in drug resistance of fungi. On the other hand, considering the importance of mitochondria in numerous cellular tasks, it is not surprising that mitochondrial pathways have been considered important targets for developing fungicides (Hahn, 2014; Mosbach et al., 2017; Young et al., 2018; Carmona et al., 2020; Medina et al.).

Fungal mitochondria harbor their own genome (mitogenome), with genetic codes different from the nuclear genome. The mitogenome is responsible for the proper functioning of the cells and stability of nuclear genome. On the other hand, to sustain their functions and integrity, mitochondria require communication with the nuclear genome (Kaniak-Golik and Skoneczna, 2015). In fungi, the majority of mitochondrial proteins are encoded in the nuclear genome, produced in the cytosol as precursors, and imported into the mitochondria (Bolender et al., 2008). The *S. cerevisiae* mitochondrial proteome includes around 1,000 proteins, while 438 have been identified in *N. crassa* using proteomic studies (Ambrosio et al., 2013).

The typical fungal mitogenome of Ascomycota and Basidiomycota (dikarya fungi) contains 14 protein-coding genes (atp6, 8, and 9; cob; cox1– 3; and nad1– 6, 4L), the rnL and rnS genes, and usually from 20 to 31 of trn genes. The *R*ps3 gene encoding the ribosomal protein S3 can be also be found in many fungal mitogenomes. Fungi show variation in gene content. For example, mitogenomes of yeasts (family Saccharomycetaceae) lack and genes (Freel et al., 2015). In addition, a patchy distribution of the rnpB gene encoding the RNA subunit of the mitochondrial RNAse P is also frequently observed. In Ascomycota, mitochondrial genes are generally encoded in one strand, but in both strands in Basidiomycota (Zardoya, 2020).

Nearly all mechanisms responsible for maintaining the nuclear genome integrity, such as mismatch repair, base excision repair, and double-strand break repair via homologous recombination or the non-homologous end-joining pathway, also occur to maintain mtDNA stability (Kaniak-Golik and Skoneczna, 2015). In fungi, the mutation rate in mtDNA is usually lower than in nuclear genomes (De Chiara et al., 2020). However, it is worth noting that the mutation rate in mtDNA appears to be dependent upon the fungal group studied. For example, in plant pathogenic *Rhynchosporium* species the mutation rate in mtDNA was proved to be higher than in nuclear genomes (Torriani et al., 2014), however, the mechanisms underlying this variation remain unclear. It has been suggested that the higher mutation rate of mtDNA could be due to poor effectiveness of the mtDNA repair system due to the nearby production of reactive oxygen species (Mendoza et al., 2020). Mitogenomes can display increased variation due to gene rearrangements caused by mitogenome recombination (Aguileta et al., 2014). Fungal mitogenomes may also differ in gene order and composition, pseudogene content and tandem repeats in intergenic regions (Aguileta et al., 2014; Fonseca et al.). Among the other explanations of mitogenome variability in fungi are double-stranded RNA elements and the frequent mobility of present self-splicing introns (Wu and Hao, 2019; Mendoza et al., 2020).

The most frequently encountered introns in fungal mitogenomes are group I introns that can encode homing endonucleases (HEGs) with either LAGLIDADG or GIY-YIG motifs. Intron encoded proteins catalyze intron mobility and have been shown in some instances to act as maturases enhancing the splicing of introns (Hausner, 2003). Distribution of these introns is irregular among different lineages and their irregular distribution has also been observed at the strain level. On the other hand, the same introns and associated HEGs may also be present in mitogenomes of evolutionarily distant lineages (Hausner, 2003). Their irregular distribution in fungi is often explained with “early intron” or “late intron” evolutionary contradicting hypotheses. The first one supports that introns were introduced to eukaryotes at an early stage of their evolution, and a general evolutionary process dominated toward the loss of introns (Goddard and Burt, 1999). The second hypothesis suggests more recent intron expansion even between diverse species through horizontal transfer (Gonzalez et al., 1998). Both hypotheses could be proven based on the most recent pan genomic approach, which indicated an intron-rich ancestor of fungi as well as the existence of intron-late events through a range of recombinational events that resulted from both vertical and horizontal gene transmissions (Megarioti and Kouvelis, 2020). Although mitochondrial introns have been viewed by many as neutral elements (Goddard and Burt, 1999) recent studies are suggesting some introns may impact mitochondrial gene expression (Rudan et al., 2018), virulence in some fungal pathogens (Baidyaroy et al., 2011; Medina et al.), and resistance to certain fungicides (Cinget and Bélanger, 2020). With regards to fungi pathogenic toward humans, as group I introns are not found in mammalian genomes, they are starting to be explored as fungal specific druggable RNA targets (Gomes et al., 2018).

Fungal mitogenomes are usually represented as circular molecules, although linear versions with defined telomeric ends, and versions existing as linear concatemers have been reported (Bullerwell and Lang, 2005; Valach et al., 2011), and they can range in size from 12.055 bp to 500 kbp (James et al., 2013; Liu et al., 2020). The size variation is in part due to introns, intergenic spacers, plasmid insertions, partial duplications, and in some instances the proliferation of repeats (Hausner, 2003; Liu et al., 2020). Patterns of variation in mtDNA provide genetic information that is often sufficiently variable for comparative genomics as well as for evolutionary studies of fungi. The use of genomic data has greatly promoted the development of taxonomy and phylogenetic relationships of fungi, leading to the taxonomic revisions and the identification of new species. However, it is worth to note that despite molecular dating, some of fungal lineages still hold an uncertain phylogenetic position (Naranjo-Ortiz and Gabaldón, 2020). Characterization of mitogenome patterns may help to resolve these questions. Some recent examples of new phylogenetic insights were derived from studies in nematode-trapping fungi (Zhang et al.), the commercialized edible mushroom *Hypsizygus marmoreus* (Wang et al., 2019), and ectomycorrhizal fungi from the genus Rhizopogon (Li et al., 2019). It is worth noting however, that estimating phylogenetic relationships of fungi based on both mitogenome and nuclear phylogenies can be difficult due to their highly discordant evolutionary trajectories (De Chiara et al., 2020).

In population genetic studies on fungi, mitogenomic analyses may give different results from nuclear genome analyses, indicating nucleus and organelle may evolve via different trajectories. Wolters et al. (2015) revealed that the distribution of mitochondrial introns in *S. cerevisiae* had population-specific profiles. However, further work by De Chiara et al. (2020) showed that despite high mtDNA polymorphism, mitochondrial population structure poorly reflects the clustering based on nuclear data, which indicated that in yeasts both nuclear and mitochondrial genomes are affected by outbreeding. The limitations of mitogenomics in population surveys have been recently underlined in studies on plant pathogenic Fusaria. Comparative analysis of strains from worldwide field populations did not reveal a link between mitogenome variation and the geographic origin of the populations mostly due to high sequence conservation and ancestral origin of introns and associated HEGs (Kulik et al.).

Mitogenomics shows promise in the field of fungal diagnostics. Efficient detection of fungi is difficult due to the inefficient recovery of genomic DNA for PCR, mostly due to the very low and uneven distribution of fungal biomass in tested samples and difficult to disrupt chitinous cell wall (Bilska et al., 2018). Markers developed on the basis of mitochondrial sequences provide a highly sensitive detection of fungi due to the multi-copy nature of mitogenomes. It has been also noted that mitochondrial DNA may be useful in deciphering cryptic species diversity (Kulik et al., 2020). However, development of novel diagnostic assays requires their evaluation in terms of uniformity and specificity against a test panel of different target and non-target fungi. This requires a continuously updating of knowledge on both fungal taxonomy and mitogenome variation. Continued investment in sequencing of both nuclear and mitochondrial genomes is necessary to open up dimensions for improved diagnostics, which should be consistent with the actual taxonomic/natural classification of fungi.

Mitogenomics can also bring new insights into the mechanisms on mitochondrial functioning, fungal pathogenicity, virulence and drug resistance. Fungi, still belong to a group of largely unexplored microorganisms in terms of integrative proteomic and genomic approaches for understanding mitochondrial processes (Ambrosio et al., 2013; Medina et al.). Today, there are a lot of open questions regarding the mitochondrial proteome and its role in the dynamic modulation of these organelle (Ambrosio et al., 2013; Mendoza et al., 2020). Mitochondrial inheritance has been explored in only a few fungal species (*S. cerevisiae, Cryptococcus neoformans, Microbotryum violaceum*, and *Ustilago maydis*) and these studies provided evidence for a large variation in inheritance mechanisms (Mendoza et al., 2020). We believe that the outlined knowledge gaps that exists in the field of fungal mitogenomics will stimulate the posting of additional questions that will promote research on the diverse cellular functions and roles of mitochondria in various fungal systems. Deciphering molecular mechanisms underlying these processes requires an integrative approach (mitogenomics, transcriptomics, and proteomics) combined with the knowledge on mitogenome structure and organization.

We are very proud of this special issue on the “Significance of Mitogenomics in Mycology” and would like to thank all contributors for submitting their cutting-edge research to it. All readers we wish a pleasant update on the possibilities and impossibilities of fungal mitogenomics in a varied range of fungal species.

## Author Contributions

All authors listed have made a substantial, direct and intellectual contribution to the work, and approved it for publication.

## Conflict of Interest

The authors declare that the research was conducted in the absence of any commercial or financial relationships that could be construed as a potential conflict of interest.
